# Source-Sink Relationships in Crop Plants and Their Influence on Yield Development and Nutritional Quality

**DOI:** 10.3389/fpls.2018.01889

**Published:** 2018-12-20

**Authors:** Millicent R. Smith, Idupulapati M. Rao, Andrew Merchant

**Affiliations:** ^1^School of Life and Environmental Sciences, Faculty of Science, Sydney Institute of Agriculture, The University of Sydney, Sydney, NSW, Australia; ^2^Centro Internacional de Agricultura Tropical, Cali, Colombia

**Keywords:** abiotic stress, crop yield potential, environment, harvest index, photosynthesis, yield

## Abstract

For seed crops, yield is the cumulative result of both source and sink strength for photoassimilates and nutrients over the course of seed development. Source strength for photoassimilates is dictated by both net photosynthetic rate and the rate of photoassimilate remobilisation from source tissues. This review focuses on the current understanding of how the source-sink relationship in crop plants influences rates of yield development and the resilience of yield and nutritional quality. We present the limitations of current approaches to accurately measure sink strength and emphasize differences in coordination between photosynthesis and yield under varying environmental conditions. We highlight the potential to exploit source-sink dynamics, in order to improve yields and emphasize the importance of resilience in yield and nutritional quality with implications for plant breeding strategies.

## Introduction

Yield of many crops rarely meets its maximum potential for production. The resulting yield gap is defined as the difference between average yield at the farm gate and crop yield potential for a specific land area. Crop yield potential as described by Evans and Fischer (1999), is the yield of an adapted cultivar grown in an ideal environment where abiotic and biotic stresses are effectively controlled. These concepts allow producers and researchers to gauge disparities in production, in particular the sensitivity of yield to stresses that can be managed, and target improvements accordingly (Evans and Fischer, 1999; Lobell et al., 2009; Van Ittersum and Cassman, 2013). However, the pursuit of reducing the yield gap as an aspirational target has limitations. Yield gap is not a direct measure (Lobell et al., 2009) and is sensitive to increases in yield potential. Similarly, targeting the yield gap does not take into account the economic considerations of production such as cost of inputs or price received for product quality (Lobell et al., 2009; Fischer et al., 2014). Nevertheless, as increases in crop yield potential are inadequate to meet future demand (Ray et al., 2013), reducing this yield gap forms an important component of multidisciplinary efforts to improve food security.

Metrics used to quantify yield are crop-specific, commonly including volume and/or weight together with plant efficiency expressed in terms such as harvest index. These metrics provide information on the characteristics that determine yield improvements under both controlled and field conditions. Despite this importance, our understanding of processes that influence harvest index is far from complete. Whilst much is known regarding the properties and processes that determine photosynthetic performance, relatively little is known regarding the dynamics and potential improvement of processes governing packaging, transport and assimilation of photoassimilates and nutrients into the developing seed. Key bottlenecks in the production of yield quantity and nutritional quality remain and represent a key focus for prioritization in addressing the yield gap and crop yield potential. Specifically, our ability to reduce the yield gap and enhance the security of food production is limited by misconceptions and knowledge gaps in: (1) the clarification of metrics to describe yield production, (2) coupling of source strength to yield production, (3) characterizing the source-path-sink transition for resources within the plant, (4) defining sink strength and its measurement, and (5) characterizing the resilience of yield quantity and quality. By revising these concepts and metrics, researchers in collaboration with producers, are able to identify mechanistically based crop traits from planting to postharvest that may be adjusted to improve overall yields and reduce yield gaps.

## Harvest Index as a Metric to Select for Improved Yield Production

The production of yield in cropping systems is a consequence of many biological processes culminating in biomass and/or seed production. Harvest index, the ratio of harvested grain to total shoot dry matter (Donald and Hamblin, 1976), is a trait that is the cumulative result of allocation of acquired resources and used in efforts to improve yields in seed producing crops (Reynolds and Langridge, 2016). Improvements to harvest index have historically increased yield potentials in major staple food crops (Hay, 1995) leading to broad economic gains for farmers. More specifically harvest index, represents the result of plant efficiency including a range of processes governing the packaging, transport and deposition of photoassimilates and nutrients into the seed. Whilst much is known regarding the processes underpinning how this is achieved (Pritchard, 2007), few studies have sought to exploit these properties to improve yield production.

Improvements in harvest index are credited with inducing large increases in yield potential in important food crops (Long et al., 2015) yet the specific mechanisms behind how this occurred are not well understood (Amthor, 2007). The fundamental basis of harvest index in seed producing crops is carbon centric and dictates that total shoot dry matter determines aboveground “sources” of photoassimilate and harvested grain represents the “sinks.” Harvest index is the proportion of biomass invested into grain (Donald and Hamblin, 1976; Gifford and Evans, 1981) and reflects the balance between source and sink (Luo et al., 2015). Measurement of harvest index does not capture the efficiency of resource investment and confounds the processes and pathways that regulate the transfer of these resources from the total shoot biomass into grain. It is therefore not surprising that harvest index correlates with various yield-related traits in important crop species though generally these are interrelated (Luo et al., 2015) further confounding the underlying mechanisms that drive increases in this important trait.

Harvest index has high heritability under both ideal and stressed environments (Hay, 1995). Conservation of the trait across multiple environments and genotypes in different crops has led harvest index to be one of the most highly studied traits in plant breeding (Unkovich et al., 2010). Much of the variation observed in harvest index values results from the diverse range of climates and soils, which are a feature of the cropping region. Factors that influence crop harvest index include the energy and protein content of seeds, long-term breeding achievements, and extreme (either hot or cold) temperatures during crop reproductive development. Finding relationships between local climate and harvest index may be used to vary harvest index temporally and spatially and this approach to plant breeding will improve carbon accounting practices. Elements of the genetic basis of harvest index have been explored in numerous studies across many species including *Brassica napus* (Luo et al., 2015), *Oryza sativa* (see, Li et al., 2012), *Glycine max* (Cui et al., 2008), and *Triticum aestivum* (Quarrie et al., 2006). However, these studies are unable to identify specific quantitative trait loci (QTLs) directly associated with harvest index. Such studies conclude that as harvest index is integrative it is therefore affected by many factors that influence source-sink dynamics (Hay, 1995; Luo et al., 2015). The integrative nature of harvest index may be one of the factors that influence this trait to be highly conserved, as multiple physiological processes influence its determination under varying environmental and genetic conditions. Despite this complexity, it is widely recognized that harvest index is an appropriate trait to target for increasing yield potentials for crop breeding activities (Amthor, 2007; Reynolds et al., 2011; Sadras and Richards, 2014). A more comprehensive understanding of harvest index and the basis upon which variations in harvest index are achieved among different genotypes would be of considerable advantage to food production systems yet the complexity of the trait inhibits its characterisation.

For our major crops, yield improvement has moderated (Amthor, 2007; Ray et al., 2013; Foyer et al., 2016). Future agricultural crop research objectives must continue to address the optimization of resource use efficiency to ensure the stability of yield (Ainsworth et al., 2012). As harvest index varies with differences in crop management (Yang and Zhang, 2010), it is likely that selecting for harvest index guarantees a high yield potential only under the environment for which it is selected. This may not necessarily lead to the resilience of yield under both ideal and stressful conditions. Harvest index may be used as a measure to indicate that more can be done to improve yields, but users must recognize that the interaction between harvest index and environmental variation is complex and may not scale accordingly with total yield. More generally, increases to harvest index are limited by both source and sink. Harvest index has a theoretical maximum and increases beyond this require additional shoot biomass (Hay, 1995). On the other side of the equation, increases in yield are limited by the number and size of grain tissue (Borrás et al., 2004; Rao et al., 2017).

To date, efforts to improve yield have been largely carbon centric although the chemical reduction of carbon into photoassimilates is unlikely to be the controlling process in plant growth, except in select systems with “luxurious” supplementation of resources such as nutrients and water (see for example, Korner, 2015). This carbon centric approach, facilitated through the use of harvest index may have led to indirect selection for alternative traits such as a crops ability to accumulate nitrogen (Sinclair, 1998). More generally, harvest index is a reflection of partitioning of photoassimilates, and nutrients throughout the plant, however, relatively little is known regarding concomitant transport and incorporation into the developing seed. Selecting for yield using integrative measures such as harvest index may have led to greater yield quantity for some crops but it has reduced the nutritional quality of that yield (see, Fan et al., 2008 for wheat), and this trend is expected to continue as atmospheric CO_2_ concentrations increase (Myers et al., 2014). Further research in this area should aim to characterize the processes determining the balance between allocation of resources to yield development and nutritional quality. Integrative traits such as harvest index are the “low hanging fruit” (Reynolds and Langridge, 2016) that allow us to see where yield improvement is required. However, the integrative nature of harvest index does not identify tangible targets for research to promote improvements in yield on the basis of chemical and/or physiological processes, nor identify potential corresponding consequences for plant growth and survival.

## Coupling Source Strength to Yield Potential

Photosynthesis is one of the most widely studied plant processes and has gained renewed focus in efforts to increase yields (Stitt et al., 2010; Foyer et al., 2017). Photosynthesis is well described and efforts to improve efficiency tend to focus on weak links with yield production or transposing different mechanisms into the pathway (for example, Long et al., 2015). Recently, support for the link between increasing photosynthesis and yield has been driven by studies performed under elevated (CO_2_) conditions which have suggested a need to increase source strength in order to improve yields (Ainsworth and Bush, 2011). Such increases in photosynthetic rates are attributable to increased substrate availability rather than photosynthetic performance with limited interpretation outside of systems supplemented with water and nutrient supply. More broadly, there is a lack of clear evidence to support the relationship between net-photosynthesis and yield beyond the concept of yield potential. Equations for yield potential outlined by Monteith (1977) describe the efficiency with which a plant intercepts light, converts intercepted radiation to biomass and partitions this biomass into the harvested product only when the given crop is grown in ideal conditions where ample nutrients, water and all biological stresses are controlled (Evans and Fischer, 1999; Long et al., 2006; Amthor, 2007).

Increases in yield potential over the past 50 years have essentially been achieved through increases to harvest index (i.e., increased partitioning of biomass into the harvest product), greater responses to additional nitrogen fertilizers and increased canopy development allowing for increased light interception (Long et al., 2006). Several authors have therefore suggested that if two out of the three components of the theoretical yield potential equation are approaching their upper limits (Lobell et al., 2009; Zhu et al., 2010), the efficiency with which light energy is converted into biomass i.e., photosynthesis, is the next target in efforts to increase yield potential (Zhu et al., 2010; Foyer et al., 2017). Little evidence supports this notion. Whilst improvements in light acquisition and utilization to drive photosynthetic performance is likely important to improving yield, no evidence exists suggesting an exhaustion in the capacity of harvest index to achieve yield gains. Beyond calculations for yield potential, correlations between crop yield and photosynthesis are weak (see, Evans, 1997) and yield is typically limited by sink capacity rather than source strength (i.e., photosynthesis) in the major crops of wheat, maize and soybean (see, Borrás et al., 2004). There is, however, some coordination between photosynthesis and yield in ideal environments where other resources are not limiting (see, Long et al., 2006 and references therein).

Studies completed in open-air elevated (CO_2_) conditions have consistently shown that a prolonged increase in photosynthesis leads to increased crop yield (Ainsworth and Long, 2005; Ainsworth and Bush, 2011). In these scenarios, carbohydrate formation may be a limiting resource controlling growth (Korner, 2015) as resources such as nutrients, light and water are in abundance. Increases in yield observed in these environments may be a consequence of increases in organ number rather than increased organ size (see, Patrick and Colyvas, 2014) suggesting that increases to crop yield (i.e., organ size x organ number) may be unrelated to carbon availability at the whole plant scale but alternatively, limited by the transport capacity of photoassimilate to small sinks (Ruan et al., 2012; Patrick, 2013). In a recent study, Gray et al. (2016) demonstrated that soybean grown under combined elevated (CO_2_) and drought did not have the same level of stimulation as provided by elevated (CO_2_) alone as the combined impact led to limitations on carbon, water and nutrient relations. Abiotic stress impacts more on growth compared to photosynthesis earlier in plant development, however, during the harvest phase the opposite occurs as the plant is able to rely on remobilization of reserves to buffer the impact of abiotic stresses. This highlights the importance of understanding the interacting factors that impact on photosynthesis and yield across a growing season as resource limitations may shift across time and space.

Several shoot and root traits contribute to superior yield and this depends on their interaction with the environmental stress. For example, plant models of isohydric (“water saving”) and anisohydric (“water spending”) have been developed for targeting genotypes according to agro-ecological zones and types of water stress. The isohydric genotype might have an advantage in the harsh environments, whereas the anisohydric genotype will perform relatively better under more moderate water stress conditions (Blum, 2015). In the anisohydric genotype, the effective use of water is relevant when there is still soil water available at maturity or when deep-rooted genotypes access water deep in the soil profile that is not normally available (Araus, 2002; Polania et al., 2017). Thus the anisohydric genotypes tend to maintain photosynthesis at low leaf water potentials during water stress.

Despite poor correlations between photosynthesis and yield under suboptimal conditions, attempts to increase the efficiency of photosynthesis are certainly valuable. However, post-photosynthetic mechanisms that may drive yield production introduce a complexity that is not encompassed by the equations used to calculate yield potential. Post-photosynthetic mechanisms for photoassimilate and nutrient transport are likely affected by prevailing environmental conditions. For instance, a large study of wheat genotypes (Lopes and Reynolds, 2012) showed a correlation between yield and prolonged flag leaf photosynthesis only under abiotic stress (drought and/or heat). Conversely, Muller et al. (2011) examined an uncoupling between photosynthesis and growth under drought conditions. Combined, these observations suggest that environmental conditions impact on the relationship between photosynthesis and yield and that these processes are not well understood. Such observations may be attributable to ontological differences and variances in determinacy coupled with the onset of stress conditions. For photosynthesis, efforts to understand responses to changing environmental conditions have allowed for manipulations that increase photosynthetic efficiency under fluctuating environmental conditions. For example, Kromdijk et al. (2016) altered photoprotection mechanisms in *Nicotiana* thus increasing photosynthetic efficiency leading to projected increases in biomass productivity by 15%. However, as current models relating photosynthetic rates to biomass production do not fully consider post-photosynthetic mechanisms and their fluctuations in response to environmental variability, it is difficult to predict the tangible impact of this change on yield.

## Characterizing the Source-Path-Sink

Despite a functional understanding of how photoassimilates are packaged and transported to sink tissues, characterizing the activity of post-photosynthetic processes that exhibit governance over yield development is rare. It is understood that only 2–4% of available radiation is converted into growth (Zhu et al., 2010) and typically that 50–80% of photoassimilates from a single mature leaf are transported into the phloem (Ainsworth and Bush, 2011). However, we have less of an understanding about the influence of changing environmental conditions on the export of carbon from leaves and import to sinks, partitioning between heterotrophic tissues and remobilization of carbohydrates into reproductive structures. To capitalize on recent advances that have improved photosynthetic efficiency, developing an understanding of the way energy and nutrients move through a plant and into a developing seed is key to ensuring the efficiency of yield production, particularly under abiotic stress.

Long distance transport is primarily achieved via the phloem stream. This link between export of sugars from leaves and the corresponding demand by sinks has been examined as a possible target for improving plant productivity (Ainsworth and Bush, 2011; Lemoine et al., 2013; White et al., 2016). Loading of metabolites into the phloem, unloading and transport are likely central mechanisms influencing yield under stress conditions and has been shown to possess flexibility according to physiological status (Turgeon, 1996). Metabolism and storage of photosynthate is tightly regulated at specific points in the pathway between source and sink (Bihmidine et al., 2013) and partitioning is thought to be a major determinant of yield. In order to appropriately target improvements in these processes, it is pertinent to consider our current understanding about the impact of environmental conditions on both the source and the sink, as well as the path between the two, as the exact mechanisms that impair transport are not well known (Lemoine et al., 2013).

**FIGURE 1:**
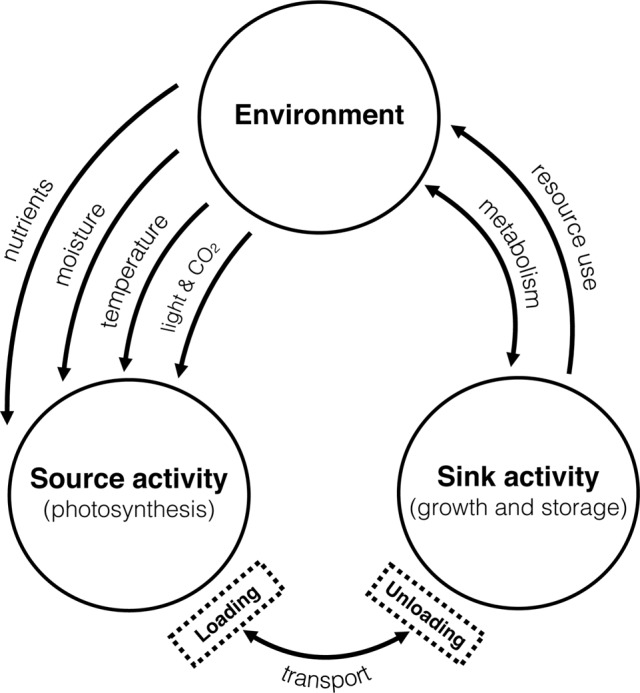
Major influences of changing environmental conditions on the relationship between source-sink dynamics. Adapted from (Korner, 2015).

Transfer of materials from source to sink is controlled by a highly regulated signaling network elicited by resource availability (Paul and Foyer, 2001; Rossi et al., 2015). Despite the importance of such a relationship, the mechanistic basis for this regulation is poorly described. In the broadest sense, it is thought that the source-sink relationship is impacted by the environment which drives source activity (photosynthesis) and consequently increases sink activity (tissue growth and storage) (see, Korner, 2015). Less considered is the influence of sink strength in this relationship and its capacity to influence source activity. The relationship is complex, and consideration must begin to focus on the dynamic nature of the network, both for source and sink strength to fully comprehend the plasticity of yield development, particularly under changing environmental conditions where elements such as carbon, nitrogen and water govern the fluxes and hence source-sink dynamics (see Figure 1).

## Defining and Measuring Sink Strength

In many cases, particularly for agricultural activities, the net cumulative result of sink strength under a given source availability is yield. Whilst the relationship between sources and sinks is undoubtedly complex, the definition typically given for sink strength is:

(1) sinkstrength=sinksizexsinkactivity

where; sink size is the total biomass of sink tissue (g), and sink activity refers to the specific uptake rate of the resource in mol g^-1^ s^-1^ (White et al., 2016). Ambiguity surrounding sink strength occurs due to an inability to directly measure (quantify) it and lack of understanding of the processes that drive sink activity. Given the number of factors that relate to sink activity (growth, metabolism), it is incongruous that sink size has a proportional influence on sink activity over sink strength as represented in Eq. 1. Whilst it is recognized that sink size has some influence over total metabolic activity, rates of metabolism vary according to ontological and tissue development. Understanding the processes and conditions governing changes to sink strength, along with improvements in technology that allow for the direct measurement of sink demand will lead to greater accuracy in the way sink strength is described. Improving the definition of sink strength will also allow for greater consistency in the literature and ensure that researchers are implementing a useful, though inevitably complex definition.

Measuring sink strength is difficult. For provision of photoassimilates, source activity can be well characterized by measurement of net photosynthetic rate. However, due to the complexity of sinks, measurement is typically confined to a quantification of sink size, typically via the removal of sink tissue, along with some measure or estimate of sink activity. Issues with this technique of mass balance measurement and others such as isotope labeling have been raised regarding the definition and measurement of sink strength (see, Farrar, 1993). In concluding the discussion, Farrar (1993) suggests that measurement of sinks should incorporate the transport system and sources. In essence, the ability to explore the complexity of sinks in the context of the whole plant without altering the system requires non-invasive technologies. Despite advances in phenotyping technologies (Rascher et al., 2011; Dhondt et al., 2013; Furbank et al., 2015), up until recently sink strength viewed in terms of carbon demand by individual sinks had still not been measured. This has prevented the exploration of questions that have interested researchers since the early discussions surrounding sink strength including the abortion of sinks and a full exploration of fluxes between sources and sinks under varying environmental conditions (see, Farrar, 1993 and references therein).

Plants aim to maintain a consistent supply of carbon and mineral nutrients to support metabolism and growth. To do this, photoassimilates are stored as starch, used directly for metabolism or synthesized into non-reducing sugars for export to sinks (Stitt et al., 2010). Distribution is thought to be determined by a sinks ability to accept photoassimilates which is dependent on the capacity to metabolize or store sugars for use. Despite the importance of partitioning on yield volume, we have just begun to understand the mechanisms responsible for the distribution of photoassimilates throughout the plant (Braun et al., 2014). Underlying partitioning is a complex signaling network involving both physical and chemical signals that play an important role in communicating sink demand, sanctioning transport and influencing overall source-sink activity (see, Bihmidine et al., 2013). Further understanding of allocation and partitioning processes on a whole plant level may enhance yield potentials by reducing photosynthate partitioned to other areas and allocating this carbon to yield. In doing so, care must be taken to ensure that this does not impact on essential aspects of plant function or reduce strategies that plants can employ in response to changes in environmental conditions.

Manipulations of sink dynamics in experimental work (see, White et al., 2016) have demonstrated both increased (Arp, 1991; Eyles et al., 2013) and decreased (Ainsworth et al., 2003; Ainsworth and Long, 2005; Ainsworth and Bush, 2011) photosynthetic rates along with changes to signaling pathways (Nunes et al., 2013). Increased partitioning to reproductive tissue has been concomitantly selected for during the breeding of *Phaseolus vulgaris* (L.) (Cuellar-Ortiz et al., 2008) suggesting that it plays an important role in the resilience of yield to abiotic stress. Nevertheless, it is unclear how the distribution of photosynthate, particularly partitioning between reproductive sinks, may occur. For instance; is carbon provided to the sink that has the highest resource demand or is it provided equally to all reproductive tissues at the same developmental stage? Do photosynthates take the shortest transport pathway between source to sink? And importantly, what implications may this lack of understanding surrounding partitioning have on the way we consider yields, particularly changing availability of resources on yield quantity and nutritional quality?

The realized movement and metabolism of sugars along the path between source and sink ultimately determines yield for which some elements of this system are well described. Movement or “loading” of photoassimilates from leaf tissues into the phloem pathway is likely an important rate-limiting step for photoassimilate movement therefore an important candidate process for improvement of transport rates. The main mechanisms for phloem loading that exist in plants are, apoplastic loading and symplastic loading (for a “comprehensive picture of phloem loading strategies” see, Rennie and Turgeon, 2009). The predominant mechanisms employed are thought to be species specific although evidence is emerging for multiple mechanisms functioning in the same plant (Rennie and Turgeon, 2009). It is likely that plants are flexible in their mode of phloem loading and alter mechanisms across development and in response to biotic and abiotic stress (see, Braun et al., 2014). The capacity for phloem loading depends upon the transport mechanism. Sucrose transporters function in apoplastic loading whereas in symplastic loading, plasmodesmal conductance for the polymer trapping mechanism are determined by catalytic interconversion of sucrose into raffinose family oligosaccharides (RFOs). The capacity of phloem loading impacts on the relationship between source and sink. As described by Ainsworth and Bush (2011); “if sink demand is high, sucrose levels are low and transcription is high. If sink demand drops, export slows and sucrose builds up and down-regulates symporter transcription and abundance. As phloem loading capacity drops, carbohydrate then builds up in the mesophyll and photosynthesis is down-regulated.” The dynamic feedback between carbohydrate utilization by the sink and production by the source clearly identifies a framework for demand-driven production by alleviating sugar mediated repression of photosynthesis (for potential strategies see, Ainsworth and Bush, 2011; Rossi et al., 2015).

Rates of phloem unloading are an important component in this framework and offers a further point of potential regulation of photoassimilate movement. Regulation and mechanisms of phloem unloading vary between species, developmental stage and sink function (Werner et al., 2011; Braun et al., 2014). While there are no direct measures of phloem loading and unloading Patrick (2013) cites, “considerable indirect evidence” that phloem unloading capacity is exceeded by photosynthesis and phloem loading particularly under optimal conditions (Voitsekhovskaja et al., 2009). Thus, using the high pressure manifold model as proposed by Fisher (2000), “resource partitioning between sinks is finely regulated by their relative hydraulic conductance of plasmodesmata linking sieve element/companion cell complexes with the surrounding phloem parenchyma cells” (Patrick, 2013). Within this model, transporters play a role in moving and partitioning photoassimilates from sources to sinks. There are numerous transporters that have been found to aid in this process. As recently demonstrated by Wang et al. (2015) in transgenic rice, enhancing transporters is another mechanism to potentially increase yields. Expanding our research into the capacity for transport and a sinks ability to accept photoassimilates is vital to ensure the resilience of yield.

## Resilience of Yield Quantity and Nutritional Quality

Improving the resilience of yield production differs to that of maximizing yields and is of great importance to food production. Depending on the agricultural context, the importance of resilience in yield production may outweigh that of maximization through the valuation of risk mitigation. Similarly, nutritional quality may be of great importance under commercial or subsistence farming scenarios. While historically, there is a strong background to understand and describe the stability of yield (Finlay and Wilkinson, 1963; Becker and Leon, 1988) under fluctuating environments; and the inverse relationship of stability, phenotypic plasticity, which describes the range of phenotypes produced by a single genotype under such environments (see, Sadras and Richards, 2014; Grogan et al., 2016; Sadras et al., 2016) currently there is no systematic approach to quantify the resilience of yield and nutritional quality under abiotic stress conditions in plant improvement programs.

**FIGURE 2:**
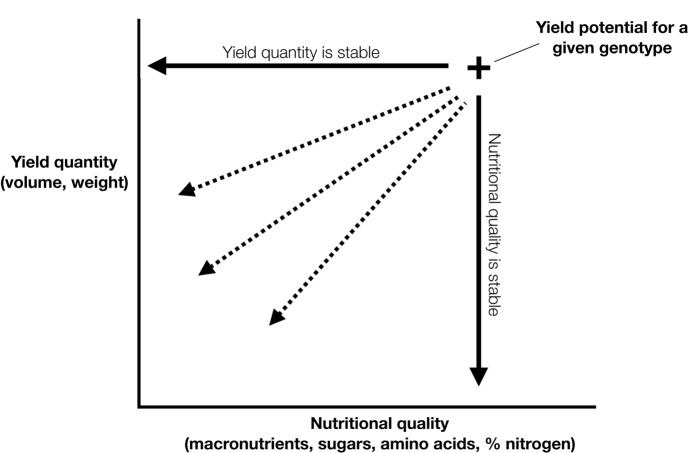
Yield quantity and nutritional quality are interrelated determining the yield potential of a particular genotype.

Resource availability undoubtedly impacts growth, yield and nutritional quality and in turn is influenced by the interaction between sources and sinks through transport pathways. Plants respond quickly to stress by altering physiology or morphology in order to survive, often enhancing reproductive fitness and yields in stressful environments. Responses and impacts of abiotic stress such as drought and nutrient deficiency have been well studied for their influence on yield development. Nutritional quality may not necessarily be flexible under varying resource availabilities. As supply of water and nutrients changes, plants may be predisposed to a set nutritional content for individual seeds, guided by a threshold for reproductive viability before then allocating resources to the next seed. Under these circumstances, nutritional quality is less coupled with resource availability. Despite this lack of understanding, the resilience of yield production and nutritional quality remain largely uncharacterised. The interconnectedness of nutritional quality and yield means that changes to one may influence the other (Figure 2). Measurement of nutritional quality and yield quantity under a range of environmental conditions may be used as a framework for phenotyping a given agricultural crop.

For agricultural production, yield is typically expressed on a volumetric or gravimetric basis, which does not directly reflect nutritional content. Given a particular environment and genotype, yield quantity and nutritional quality will fluctuate. For plants, nutrition and energy are related, not only through the highly regulated process of photosynthesis, but also through a broader scope of plant processes. For instance, the volume of soil occupied by a crop’s root tissue strongly influences the amount of nutrients available for the function and development of plant tissues (Poorter et al., 2012), and developing seeds in particular. Understanding the way energy and nutrients move through a plant and into a developing seed is key to ensuring the efficiency of yield production. Based on this framework, it is possible that nutritional quality will reflect the environmental conditions in which the plant is grown (Figure 2). While previous research has focused on the nutritional content of grain in response to an environmental change, for instance elevated (CO_2_) (Myers et al., 2014) there has been little research on the resilience of grain nutritional quality under ideal or stress conditions.

The molecular and physiological events leading to seed formation are far from understood (Abid et al., 2009). Understanding the processes involved in seed development such as the resilience of developmental processes to changes in resource supply is essential to improve overall crop yields (Bennett et al., 2011). In particular, it is important to understand the growth stages, such as seed development, where a plant may be vulnerable to stresses that will impact upon reproductive development (Kranner et al., 2010). While we have an understanding regarding the functional changes that occur throughout seed development, quantification of photoassimilate deposition into a developing seed and the consequences this might have for its final nutritional content are less characterized. This gap in our understanding has both spatial and temporal dimensions. For instance, the prioritization of individual grain development both along the nodes of the plant and throughout development requires further exploration through detailed phenotypic characterisation of individual seed development.

Plants respond to stress in a number of ways to maintain yield (Chaves and Oliveira, 2004) particularly with respect to the determinacy of growth. For instance in many crops, remobilisation of photoassimilates can occur during drought stress to compensate for reduced photosynthetic rates partitioning carbohydrates stored in stems into soluble sugars for remobilisation into grains (Blum, 2005). The objective of plants in developing yield differs to that of the nutritional needs of humans. In some cases (not all), attempts to improve nutritional quality for human consumption seeks to work against processes forged under evolutionary pressures for reproductive viability. This disconnect is particularly important under abiotic stress conditions where shifts in resource use further widen the gap between plant nutritional quality and human nutritional requirements. A sustainable supply of protein, vitamins, macro and micronutrients is a major driver for health and wellbeing in communities. Aiming to maintain a consistent, resilient supply of yield from plants working with the capacity to adapt to stress while maintaining high nutritional quality is vital. In addition, the complex nature of plant function demonstrates a remarkable ability to adjust to changing resource availability, but this may have implications on other plant processes or properties for instance nutrient acquisition. For example; severe drought, may alter plant properties such as root system architecture and growth (exploration of the soil both positively and negatively). In a well characterized example, chickpea yield was found to have a greater nutritional quality under drought stress as a result of increased accumulation of soluble sugars, amino acids and proteins to the grain (Behboudian et al., 2001). Such responses may be explained by changes in architecture subsequently influencing access to essential nutrients such as phosphorus or calcium when rooting depth increases in the search for more water (Chaves et al., 2003). Drought stress therefore leads to a change in the composition and concentration of nutrients potentially altering the nutritive quality of yield under stress conditions. The magnitude of these effects is likely to have an impact on nutritional quality of food production highlighting the need to incorporate yield quality and its stability under changing climatic and edaphic conditions into crop pre-breeding research.

## Conclusion

It is clear that 21st century agricultural production faces many challenges. Traditional focus on maximizing yield and the development of superior genotypes for ideal growth conditions is unlikely to provide adequate nutrition for a growing population under a more variable climate. The concepts of resilience and nutritional quality need to be incorporated into breeding strategies to provide adequate tools for farmers across the development spectrum. At the plant scale, focus must be placed on components that enhance plant efficiency and yield production under a range of climatic conditions and resource availabilities. We include here, suggested additions to the conceptual framework for yield production and proposed practical solutions as summarized in Table 1. Considerable scope exists for improvements in our understanding of post-photosynthetic processes that determine yield, notably the fundamental property of sink demand, determinacy of sink development and the corresponding influence of these processes on source function and ontological development. The clarification and revision of these concepts for improving plant efficiency will enable researchers and producers to provide a more mechanistically based research for development approach to achieving the objectives of plant breeding on a background of enhanced phenotyping capacity.

**Table 1:** Concepts, limitations and proposed solutions that may be adopted to improve plant efficiency, yield and nutritional quality under a range of environmental conditions.

Concept	Limitation	Proposed solution	Adoption in crop research
Harvest index (HI) as a metric for yield production	• Limited understanding of specific mechanisms behind HI improvement • Doesn’t capture efficiency of resource investment • Doesn’t necessarily relate to yield resilience if selected under ideal conditions • May alter nutritional quality	• Use HI with the understanding that it is an integrative trait which makes it difficult to target specific attributes for breeding • Consider the implications of selecting for HI on plant resource use and nutritional quality	•Luo et al. (2015) explored the complexity of HI in *Brassica napus* L. using association mapping
Coupling source strength to yield potential	• Post-photosynthetic mechanisms lead to complexity that isn’t included in yield potential equations • Differences in coordination between photosynthesis and yield under varying development stages and environmental conditions	• Increase photosynthetic efficiency under changing environmental conditions • Incorporate mechanisms post-photosynthesis on yield potential predictions	• Increasing the photosynthetic efficiency is the focus of the Realizing Increased Photosynthetic Efficiency (RIPE) project (Long et al., 2015). This has involved research in cassava (De Souza et al., 2017) and soybean (Srinivasan et al., 2017)
Definition of sink strength	• Sink size doesn’t have a proportional influence on sink strength as sink activity can change	• Sink strength definition should incorporate development, a measurement of sink demand to reproductive tissues and, metabolism • Recognition that sink strength is contextual	• The link between source and sink has been identified as a potential target to improve productivity by Ainsworth and Bush (2011)
Measurement of sink strength	• Sink strength cannot be measured beyond calculations of sink size	• Use non-invasive tools to measure sink demand/strength	• Preliminary methodologies have been described (for example, Windt and Blumler, 2015)
Resilience of nutritional quality and quantity	• No systematic approach to quantify yield resilience in plant improvement programs	• Research resilience mechanisms under a range of environmental conditions and corresponding flexibility in nutritional quality	• Traits that underpin the stability of yield under varying environmental conditions have been explored in wheat (Grogan et al., 2016) • Fluctuations in nutrient content have been identified for a range of crop species under elevated (CO_2_) (Myers et al., 2014) and in chickpea grown under drought stress (Behboudian et al., 2001)

## Author Contributions

MS and AM conceived, designed and wrote the manuscript. IR contributed expertise in crop physiology and plant nutrition.

## Conflict of Interest Statement

The authors declare that the research was conducted in the absence of any commercial or financial relationships that could be construed as a potential conflict of interest.
